# The Research Landscape of Ferroptosis in Cancer: A Bibliometric Analysis

**DOI:** 10.3389/fcell.2022.841724

**Published:** 2022-05-25

**Authors:** Guoli Li, Yumei Liang, Hongyu Yang, Weiru Zhang, Tingting Xie

**Affiliations:** ^1^ Department of Nephrology, Hunan Provincial People’s Hospital, The First Affiliated Hospital of Hunan Normal University, Changsha, China; ^2^ Changsha Clinical Research Center for Kidney Disease, Changsha, China; ^3^ Hunan Clinical Research Center for Chronic Kidney Disease, Changsha, China; ^4^ Department of General Medicine, Xiangya Hospital, Central South University, Changsha, China; ^5^ International Collaborative Research Center for Medical Metabolomics, Xiangya Hospital Central South University, Changsha, China; ^6^ National Clinical Research Center for Geriatric Disorders (Xiangya Hospital), Changsha, China

**Keywords:** ferroptosis, cancer, bibliometric analysis, citespace, VOSviewer, visualization

## Abstract

**Background:** Ferroptosis is a novel mechanism of programmed cell death coined in 2012, which has been found to play important roles in human health and disease. In the past decade, ferroptosis research has seen booming growth worldwide. The aim of this study was to visualize the scientific outputs and research trends of ferroptosis in the field of cancer.

**Methods:** The raw data of publications were retrieved from the Web of Science Core Collection on 19 December 2021. The information on the impact factor (IF) and Journal Citation Reports (JCR) division were obtained from the website of Web of Science. Two kinds of software (CiteSpace and VOSviewer) were used to perform visualized analysis.

**Results:** From 2012 to 2021, a total of 1833 publications related to ferroptosis in cancer were identified for final analysis. The annual number of citations and publications grew exponentially over the past decade. China (1,092) and United States (489) had the highest number of publications; Central South University and Guangzhou Medical University were the most productive institutions. Daolin Tang and Scott J Dixon were the most active authors ranked by most productive and co-cited, respectively. The journals with the highest output and co-citation frequency were *Biochemical and Biophysical Research Communications and Cell*, respectively. Among the 1833 publications, four were identified with citations more than 1000 times. Six co-cited references had a citation burst duration until 2021. Analysis of keywords suggested the current research of ferroptosis in cancer clusters in 9 hotspots and newly emerging frontier may be “multidrug resistance”.

**Conclusion:** Cancer research is the major area of active research in ferroptosis. Our results provide a global landscape of the ferroptosis research in cancer from 2012 to 2021, which serves as a reference for future studies in this field.

## Introduction

Ferroptosis, a term coined fairly by Dixon et al., in 2012, is a form of programmed non-apoptotic cell death characterized by iron-dependent lipid peroxidation (Dixon et al., 2012). Since then, much attention has been attracted to the research field of biomedicine over the past decade. To date, ferroptosis has been reported in a series of human diseases, including carcinogenesis(Chen et al., 2021b), aging-related diseases(Jenkins et al., 2020; Yan et al., 2021), ischemia-reperfusion injury(Stockwell et al., 2017; Yan et al., 2022), and cardiovascular diseases(Zhang et al., 2021d). It has gradually become a hotspot and rising star in biomedical research.

Unlike other types of regulated cell death, there are three distinct biochemical hallmarks of ferroptosis: accumulation of redox-active iron, induction of lipid peroxidation, and loss of antioxidant defense (Dixon and Stockwell, 2019). Together, these events contribute to the unrestrained phospholipid peroxidation (PLOOHs), which sequentially leads to the plasma membrane rupture and initiated the process of ferroptosis (Jiang et al., 2021). Although the exact molecular mechanism of ferroptosis is not yet fully understood, several key regulators (such as glutathione peroxidase 4 and system x_c_^–^ cystine/glutamate antiporter) and pathways (such as Hippo–YAP, AMPK, and hypoxia pathways) have been convincingly established and recently reviewed (Dixon and Stockwell, 2019; Hadian and Stockwell, 2020; Chen et al., 2021a; Chen et al., 2021b; Jiang et al., 2021).

Cancer, a disease resulting from abnormal cell proliferation, has become a major public health problem worldwide (Sung et al., 2021). The balance between cell death and survival is directly linked to the development of cancer and regulated by programmed cell death mechanisms including apoptosis, necroptosis, pyroptosis, autophagy, and ferroptosis(Strasser and Vaux, 2020). Actually, the initial discovery of ferroptosis is the result of screening novel small molecule compounds for anticancer therapies(Dolma et al., 2003). Since then, cancer-related research has been extensively conducted in the context of ferroptosis. Emerging evidence implicated that the role of ferroptosis in cancer is complex and context-dependent with both promotion and suppression effects in tumorigenesis(Chen et al., 2021b). Preliminary findings suggested that triggering ferroptosis holds great potential for the development of cancer treatment including chemotherapy, radiotherapy, immunotherapy, and nano therapy (Shen et al., 2018; Liang et al., 2019; Chen et al., 2021b; Lei et al., 2021).

The term bibliometric was coined in 1969 by Alan Pritchard (Pritchard, 1969). Bibliometric analysis is a powerful tool that uses literature metrics or indicators to quantitively measure the research performance in a certain field (Deng et al., 2020; Wang et al., 2021). CiteSpace and VOSviewer are two commonly used visualizing processing tools for bibliometric analysis (Moral-Muñoz et al., 2020). A good bibliometric analysis can help researchers get the research hotspots and advances timely and comprehensively (Chen et al., 2020).In addition, compared to traditional review, the bibliometric analysis is more intuitive. Previously, several traditional reviews have described the roles of ferroptosis in cancer (Shen et al., 2018; Hassannia et al., 2019; Liang et al., 2019; Chen et al., 2021b). However, to our best knowledge, no bibliometric studies have been performed in the field of ferroptosis research in cancer.

Herein, to draw a full map of ferroptosis research trends in the field of cancer, we performed a bibliometric analysis of publication output, distribution across countries/regions and institutions, contribution of authors and journals, and the most frequently cited papers and appeared keywords. Our results provided a comprehensive understanding of development in the research field of ferroptosis in cancer, which may help future researchers.

## Materials and Methods

### Data Collection

Raw data were extracted from the database of Web of Science Core Collection. The literature search was performed within 1 day on 19 December 2021. The search formula was set as follows: ferroptosis (Topic) and “tumor*” or “cancer*” or “carcino*” or “onco*” (All Fields) and 2012–2021 (Year Published) and Meeting Abstracts or Editorial Materials or Early Access or Book Chapters or Corrections or News Items or Letters or Proceedings Papers or Retractions or Retracted Publications (Exclude—Document Types). A total of 1836 works of literature were obtained and exported for full record and cited references in the format of plain text files. Using Endnote software, three duplications were found. After removing duplications, a total of 1833 unique records remained, including 1,381 articles and 452 reviews (Supplementary Table S1).

### Visualized Analysis

CiteSpace software (Drexel University, Philadelphia, PA, United States) is a freely available Java application, which was widely used for visualizing and analyzing trends and patterns in the scientific literature (Chen, 2006). It was designed by Dr. Chaomei Chen in 2004 (Chen, 2004) and last updated on 17 January 2021 (Version 5.8.R2) (http://cluster.cis.drexel.edu/∼cchen/citespace/). Web of Science was the primary source of input data for CiteSpace. In this study, CiteSpace was used to get the map of network visualization (Countries/Regions, Institutions, Authors), the timeline view of co-occurrence (Keywords), and citation bursts (References and Keywords). The primary parameters were set as follows: time slicing (2012–2021), years per slice (1 year), selection criteria (g-index, k = 25), and pruning (minimum spanning tree, pruning sliced networks). Other parameters were set according to the CiteSpace manual for different situations.

VOSviewer software is a useful tool for constructing and visualizing bibliometric networks (van Eck and Waltman, 2010). It was developed by the Center for Science and Technology Research at Leiden University (Netherlands) in 2007 (van Eck and Waltman, 2007). The latest version 1.6.17 was released on 22 July 2021 and is free for download (https://www.vosviewer.com/). Co-authorship networks, citation-based networks, and co-occurrence networks can be created based on data downloaded from the Web of Science. In this study, VOSviewer was used to get the density map of co-cited authors, co-cited journals, and the co-occurrence of keywords.

Other information such as impact factor (IF) and Journal Citation Reports (JCR) division of journals were obtained from the Web of Science website directly on 19 November 2021. Microsoft Office Excel 2016 was used to analyze the annual publications. GraphPad Prism version 8 was used for statistical analysis. Pearson’s correlation analysis was used to investigate relationships between the annual publication numbers and annual citation numbers. A *p* value of <0.05 was considered statistically significant.

## Results

### Trends in Publication Outputs

**FIGURE 1:**
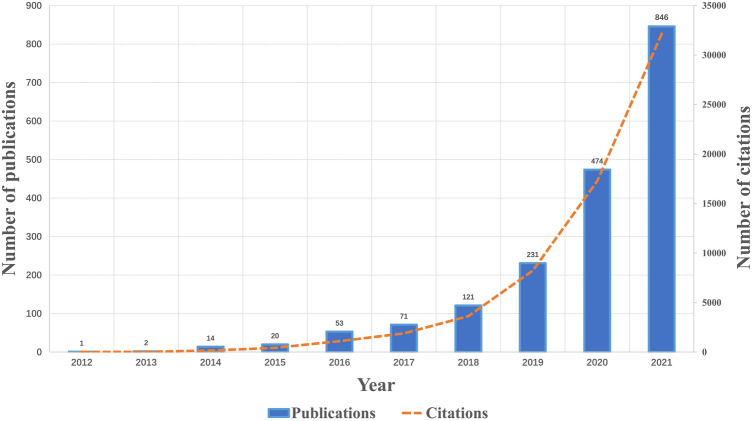
Number of annual publications and citations of ferroptosis in cancer from 2012 to 2021.

From the first paper published on 25 May 2012 to 19 December 2021, a total of 1833 publications related to ferroptosis in cancer were identified. Figure 1; Table 1 showed the annual number of publications of ferroptosis in cancer. In general, the annual number of publications increased year by year. The early stage (2012–2017) saw a relatively stable growth, with a publication counts of no more than 100 per year. While a steep growth of annual publication numbers was observed since 2018, approximately doubling every year. Up to date (19 December 2021), the publication outputs have reached over 846 in 2021. Compared to other fields of ferroptosis, the publications on ferroptosis in cancer account for most of the total publications annually (Table 1; Supplementary Figure S1). The prediction analysis indicated that over 3,500 papers will be published in 2025 (Supplementary Figure S2). As the number of citations also reflects research progress in a field (Waltman, 2016), citation analysis was performed in parallel. Similar to the trend of annual publication numbers, the annual citation numbers increased exponentially in the past decade (Figure 1). Indeed, the annual number of citations and publications was significantly correlated (Pearson *r* = 0.9994, *p*-value < 0.0001) (Supplementary Figure S3).

**TABLE 1:** The annual number of publications on ferroptosis.

	Ferroptosis in cancer			Ferroptosis in all fields		
Year	Count	% of 1,833	Citation	Count	% of 2,805	Citation
2012	1	0.05	8	1	0.04	8
2013	2	0.11	25	5	0.18	31
2014	14	0.76	168	16	0.57	183
2015	20	1.09	424	27	0.96	457
2016	53	2.89	1,111	63	2.25	1,207
2017	71	3.87	1885	96	3.42	2,102
2018	121	6.60	3,632	182	6.49	4,195
2019	231	12.60	8,228	360	12.83	9,713
2020	474	25.86	17,276	738	26.31	20,981
2021	846	46.15	32,140	1,317	46.95	39,630

### Geographical Distribution of the Publications

**FIGURE 2:**
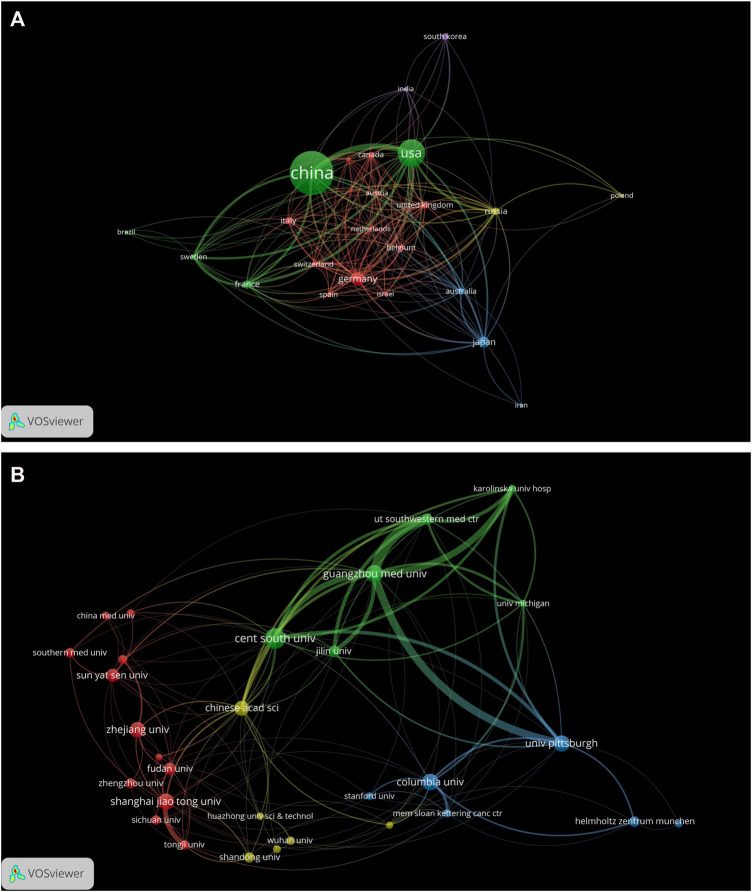
Distribution of publications from different countries/regions and institutions. **(A)** Distribution of publications from different countries/regions (N = 23, Minimum number of publications of one country/region≥10). Data from England, Scotland, and Wales were integrated into the United Kingdom; data from Taiwan and the People’s Republic of China were integrated into China. The nodes represented countries/regions and the size of the node represents the number of publications. The lines between the nodes represent cooperation relationships and the thickness of the lines was correlated with the size of collaboration. The colors in the nodes represented the clusters. **(B)** Distribution of publications from different institutions (N = 30, Minimum number of publications of one institution≥27). The nodes represented institutions and the size of the node represented the number of publications. The lines between the nodes represent cooperation relationships and the thickness of the lines was correlated with the size of collaboration. The colors in the nodes represented the clusters.

VOSviewer co-authorship analysis among the countries/regions was conducted to generate a network visualization map. Geographically, a total of 1833 articles were published from 63 different countries/regions covering five continents. Notably, Taiwan and the People’s Republic of China were merged into China; England, Scotland, Northern Ireland, and Wales were merged into the United Kingdom. To make the network clear, only 23 selected countries/regions that published at least 10 documents were visualized in Figure 2A. A complete list of documents, citations, and total link strength of the 63 countries/regions was displayed in Supplementary Table S2. Each node represented a different country/region. The size of the nodes is determined by the number of publications (The higher the number, the larger the node). The same color represented the same cluster. The line between the nodes represented the co-authorship between countries/regions (The stronger the cooperation relationship, the wider the line). The number of total link strengths reflected the total co-authorship strength between countries/regions.

As we can see from these results, there were 14 countries/regions that published only one paper and four countries/regions that published over 100 papers. Among the top 10 most prolific countries/regions (Table 2), China had the highest number of publications (1,092, 59.57%), followed by United States (489, 26.68%), Germany (151, 8.24%), and Japan (102, 5.56%). The United States had the highest number of citations (40,675), total link strength (416), and Germany had the highest number of Centrality (0.71). While Australia obtained the highest number of citations/documents (100.36) Here, high centrality (≥0.10) indicates active cooperation. Most of countries/regions cooperated with others (n = 60, total link strength≥1). The United States had the highest link strength (416), followed by China (333), Germany (226), and France (140) (Table 2). Notably, the total link strength between China and United States reached 159 (data not shown), indicating that these two countries with top publication outputs collaborated closely.

**TABLE 2:** Top 10 countries contributed to publications of ferroptosis in cancer.

Rank	Country	Year	Document	Percentage (n/1833)	Citation	Citation/document	Total link strength	Centrality
1	China	2014	1,092	59.57	25,318	23.18	333	0.06
2	United States	2012	489	26.68	40,675	83.18	416	0.23
3	Germany	2014	151	8.24	14,280	94.57	226	0.71
4	Japan	2014	102	5.56	6,945	68.09	93	0.06
5	France	2013	68	3.71	5,166	75.97	140	0.33
6	Russia	2015	51	2.78	3,162	62.00	105	0.00
7	Italy	2017	51	2.78	2,789	54.69	66	0.28
8	Australia	2016	45	2.45	4,516	100.36	78	0.06
9	South Korea	2015	44	2.40	1,479	33.61	17	0.17
10	Canada	2016	40	2.18	3,382	84.55	78	0.12

### Contributions of Institutions

A total of 1737 institutions were identified that contributed to the 1833 papers. The top 10 most prolific institutions are shown in Table 3. Also, a complete list of documents, citations, and total link strength numbers of the 1833 institutions was displayed in Supplementary Table S3. As we can see, all of the top 10 institutions came from China (n = 7) and the United States (n = 3). Central South University (92, 5.02%) is the leading institute ranked by the number of publications, followed by Guangzhou Medical University (72, 3.93%) and Columbia University (67, 3.66%). Columbia University contributed the most citations (16,996) and had the highest number of citations/document (253.67). Guangzhou Medical University had the highest number of total link strengths (287) and Zhejiang University showed the highest centrality (0.24).

**TABLE 3:** Top 10 institutions contributed to publications of ferroptosis in cancer.

Rank	Institution (Country)	Year	Document	Percentage (n/1833)	Citation	Citation/document	Total link strength	Centrality
1	Cent South Univ (China)	2014	92	5.02	3,617	39.32	201	0.23
2	Guangzhou Med Univ (China)	2015	72	3.93	7,029	97.63	287	0.00
3	Columbia Univ (United States)	2012	67	3.66	16,996	253.67	204	0.10
4	Univ Pittsburgh (United States)	2015	66	3.60	9,305	140.98	272	0.07
5	Shanghai Jiao Tong Univ (China)	2018	65	3.55	839	12.91	115	0.02
6	Zhejiang Univ (China)	2018	62	3.38	799	12.89	95	0.24
7	Chinese Acad Sci (China)	2017	57	3.11	2,292	40.21	177	0.23
8	Sun Yat Sen Univ (China)	2018	47	2.56	702	14.94	66	0.01
9	Fudan Univ (China)	2019	43	2.35	493	11.47	74	0.22
10	Ut Southwestern Med Ctr (United States)	2016	39	2.13	1,393	35.72	150	0.09

The co-authorship map of the top 30 selected institutions that published at least 27 documents was visualized in Figure 2B. The map had a total of 30 terms, four clusters, 136 links, and a total link strength of 449. Each node represented a different institution. The size of the nodes was determined by the number of publications (The higher the number, the larger the node). The same color represented the same cluster. The line between the nodes represented the co-authorship between institutions (The stronger the cooperation relationship, the wider the line). The number of total link strengths reflected the total co-authorship strength between institutions. From the visualization map, we can see that there had active cooperation among these 30 institutions within and between clusters. The top three institutions with the largest total link strength among the 1833 institutions are listed as follows: Guangzhou Medical University (n = 287), University of Pittsburgh (n = 272), and Columbia University (n = 204) (Supplementary Table S3).

### Contributions of Authors

**FIGURE 3:**
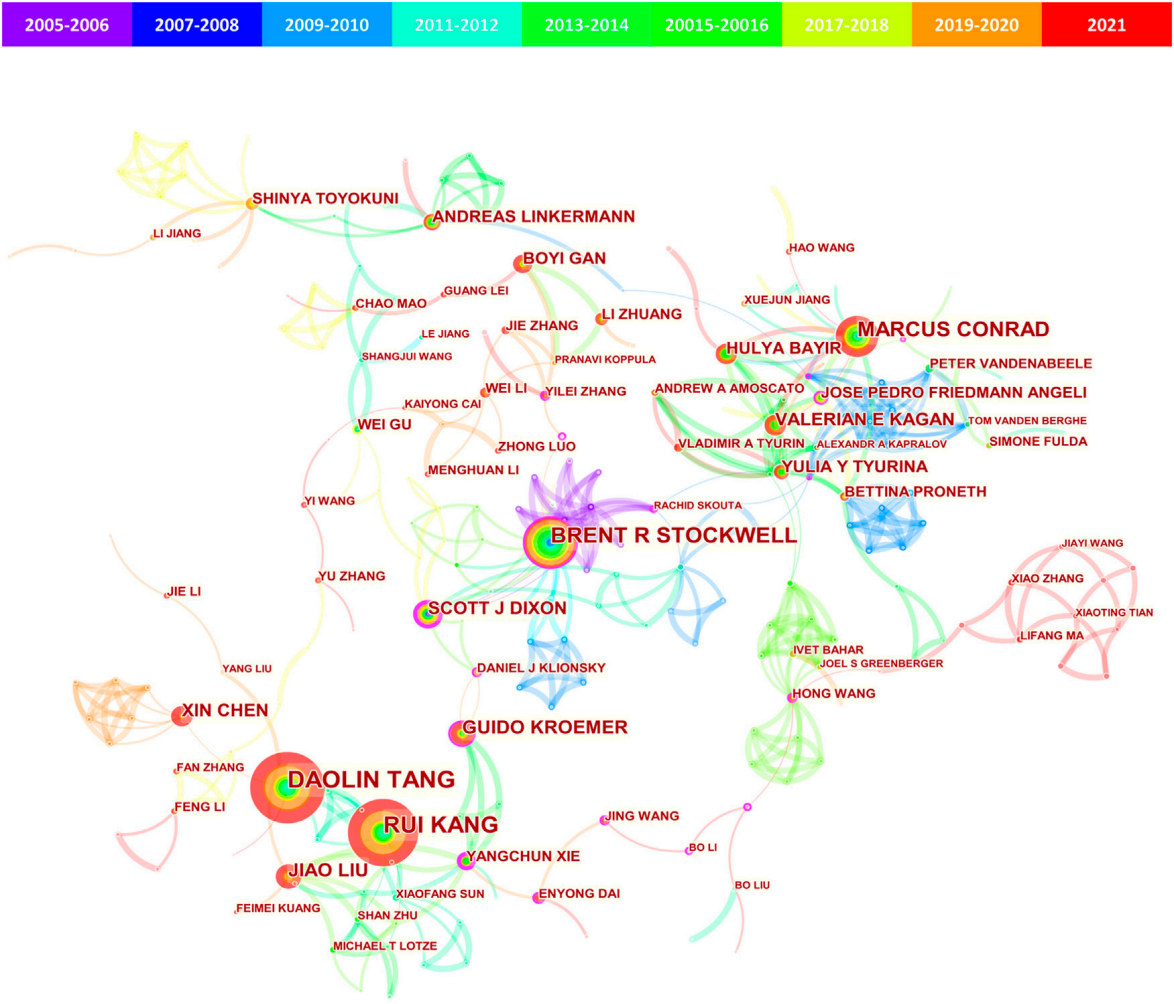
CiteSpace visualization map of authors involved in ferroptosis in cancer. The nodes represent authors (the larger circle, the higher the number of publications); lines between the nodes represented a collaboration between two authors on the same article (the wider lines, the more frequency of collaborations); the colors in the nodes represented the years, and the purple ring represented high centrality (≥0.10).

A total of 10,032 authors published literature on ferroptosis with 87 authors having papers over five(Supplementary Table S4). The author visualization map was created by Citespace (Figure 3) and the top 10 authors ranked by publication frequency and centrality were shown in Table 4. In the cooperative network map (Figure 3), the nodes represented authors (the larger circle, the higher the number of publications); lines between the nodes represented a collaboration between two authors on the same article (the wider lines, the more frequency of collaborations); the colors in the nodes represented the years, and the purple ring represented high centrality (≥0.10). As shown in these data, Daolin Tang published the largest number of papers (n = 64), followed by Rui Kang (n = 58) and Brent R Stockwell (n = 48). Both Brent R Stockwell and Scott J Dixon published earliest (in 2012) and had the highest centrality (0.20).

**TABLE 4:** The top 10 authors and co-cited authors in the field of ferroptosis in cancer.

Rank	Author	Count	Year	Centrality	Co-cited author	Co-citation
1	Daolin Tang	64	2015	0.04	Scott J Dixon	2,134
2	Rui Kang	58	2016	0.08	Wan Seok Yang	1722
3	Brent R Stockwell	48	2012	0.20	Brent R Stockwell	795
4	Marcus Conrad	38	2015	0.04	Minghui Gao	748
5	Jiao Liu	24	2018	0.06	Jose Pedro Friedmann Angeli	740
6	Guido Kroemer	23	2014	0.16	Sebastian, Doll	682
7	Scott J Dixon	21	2012	0.20	Le Jiang	525
8	Valerian E Kagan	20	2017	0.03	Lorenzo Galluzzi	468
9	Xin Chen	19	2020	0.03	Yangchun Xie	448
10	Hulya Bayir	18	2017	0.03	Xiaofang Sun	431

Co-citation was defined as the frequency with which two documents were cited together by another or more papers at the same time, providing a way to study the specialty structure of science. In this study, a total of 45,521 co-cited authors were identified with 99 co-cited over 100 times (Supplementary Table S5). The co-citation density map showed the authors (n = 46) with co-citations of at least 150 and the top 10 co-cited authors were presented in Table 4. Scott J Dixon was the most frequently co-cited author (2,134 times), followed by Wan Seok Yang (1722 times) and Brent R Stockwell (795 times). It was worth noting that Scott J Dixon and Brent R Stockwell were among the top 10 productive authors and co-cited authors.

### Journals and Co-Cited Journals

A total of 515 academic journals had published the 1833 papers on ferroptosis in cancer. The top 10 journals ranked by the number of publications were shown in Table 5, and a complete list of the 515 journals was displayed in Supplementary Table S6. Among the top 10 journals, *Biochemical and Biophysical Research Communications* (n = 54) and *Frontiers in Oncology* (n = 54) published the largest number of papers, followed by *Frontiers in Cell and Developmental Biology* (n = 52), and *Cell Death* & *Disease* (n = 51). Nine (90%) of the journals had an IF 2020 over five and seven (70%) were located in JCR (2020) Q1 region. *Biochemical and Biophysical Research Communications* had the largest number of total citations (1902 times). As shown in Table 6, the top three journals with the largest co-citations were *Cell* (co-citation: 5,342; IF 2020: 41.582), *Nature* (co-citation: 4,725; IF 2020: 49.962), *Proceedings of The National Academy of Sciences of The United States of America* (co-citation: 3,040; IF 2020: 11.205). Despite having few publications (n = 7) and a low impact factor (IF 2020: 5.157), the papers published in the *Journal of Biological Chemistry* obtained a large number of co-citations (2,882 times).

**TABLE 5:** Top 10 journals published papers of ferroptosis research in cancer.

Rank	Journal	Count	Citation	IF (2020)	JCR Category Quartile
1	Biochemical and Biophysical Research Communications	54	1902	3.575	Biochemistry & Molecular Biology (Q3); Biophysics (Q2)
2	Frontiers in Oncology	54	308	6.244	Oncology (Q2)
3	Frontiers in Cell and Developmental Biology	52	259	6.684	Cell Biology (Q2); Developmental Biology (Q1)
4	Cell Death & Disease	51	1,264	8.469	Cell Biology (Q1)
5	Redox Biology	38	1,039	11.799	Biochemistry & Molecular Biology (Q1)
6	International Journal of Molecular Sciences	36	535	5.924	Biochemistry & Molecular Biology (Q1); Chemistry, Multidisciplinary (Q2)
7	Free Radical Biology and Medicine	34	1,683	7.376	Biochemistry & Molecular Biology (Q1); Endocrinology & Metabolism (Q1)
8	Oxidative Medicine and Cellular Longevity	27	587	6.543	Cell Biology (Q2)
9	Nature Communications	26	745	14.919	Multidisciplinary Sciences (Q1)
10	Cancers	26	275	6.639	Oncology (Q1)

**TABLE 6:** Top 10 co-cited journals by papers of ferroptosis research in cancer.

Rank	Co-cited journal	Citation	Count	IF (2020)	JCR Category Quartile
1	Cell	5,342	8	41.584	Biochemistry & Molecular Biology (Q1); Cell Biology (Q1)
2	Nature	4,725	11	49.962	Multidisciplinary Sciences (Q1)
3	PNAS^a^	3,040	13	11.205	Multidisciplinary Sciences (Q1)
4	Journal of Biological Chemistry	2,882	7	5.157	Biochemistry & Molecular Biology (Q2)
5	Cell Death and Differentiation	2,394	22	15.828	Biochemistry & Molecular Biology (Q1); Cell Biology (Q1)
6	Free Radical Biology and Medicine	2083	34	7.376	Biochemistry & Molecular Biology (Q1); Endocrinology & Metabolism (Q1)
7	Cancer Research	2019	12	12.701	Oncology (Q1)
8	Nature Chemical Biology	1783	11	15.04	Biochemistry & Molecular Biology (Q1)
9	Biochemical and Biophysical Research Communications	1,697	54	3.575	Biochemistry & Molecular Biology (Q3); Biophysics (Q2)
10	Cell Death & Disease	1,551	51	8.469	Cell Biology (Q1)

a PNAS: proceedings of the national academy of sciences of the United States of America.

### Top Cited Publications and References Burst

**FIGURE 4:**
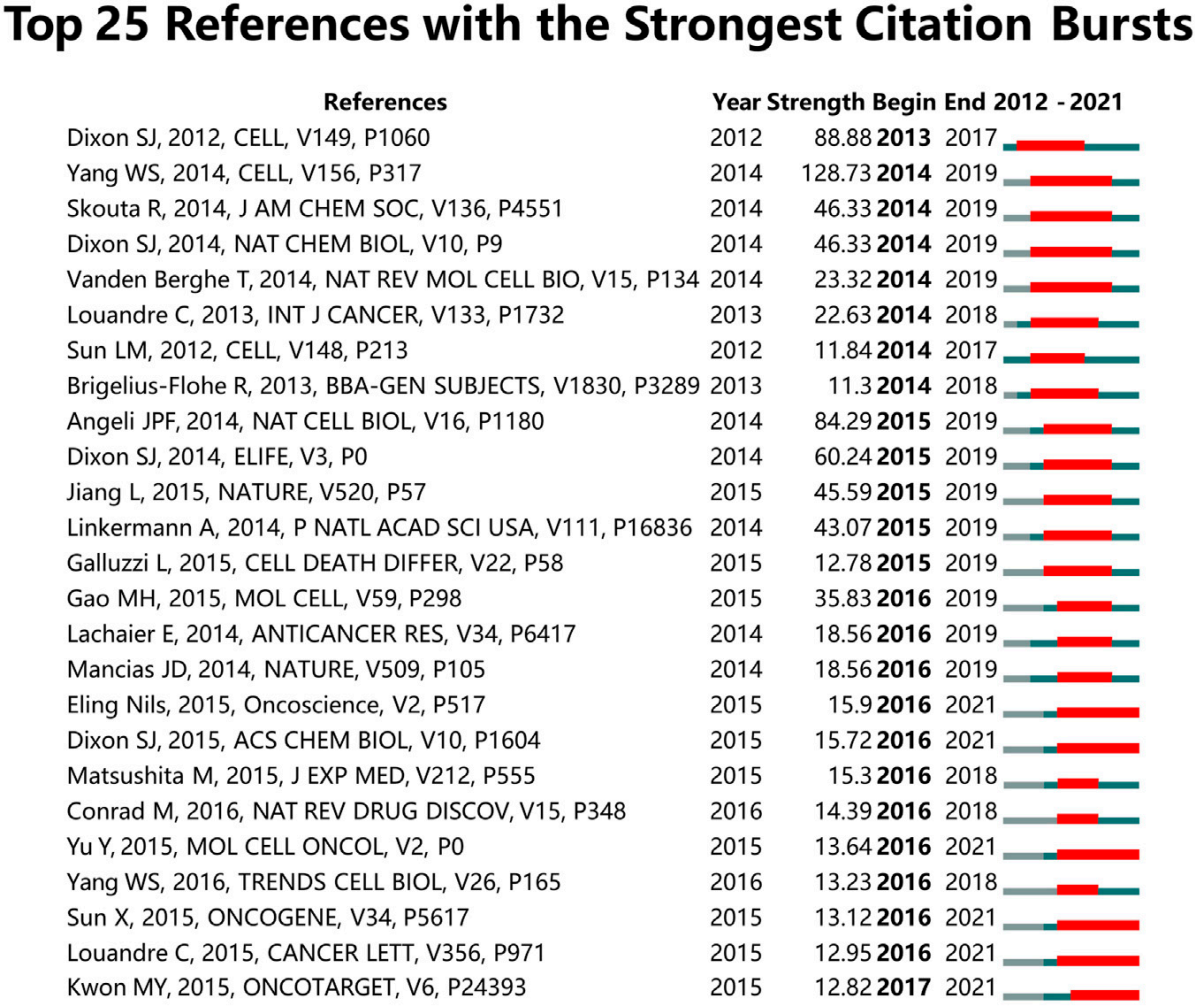
Top 25 references with the strongest citation bursts involved in ferroptosis in cancer. (Sorted by the beginning year of burst.)

A total of 20 most cited publications on ferroptosis in cancer were listed in Table 7. Of these 20 publications, fourteen were articles (70%) and six were reviews (30%). All the top 20 references were cited more than 400 times. The article (entitled Ferroptosis: An Iron-Dependent Form of Nonapoptotic Cell Death) published in 2012 by Dixon, Scott J et al. had the largest number of citations (3,009). The purpose of burst detection was to determine whether an entity had significantly increased its presence within a certain period(Kleinberg, 2003). Through CiteSpace analysis, the top 25 references with the strongest citation bursts were identified (Figure 4). The result showed the first burst of reference began in 2013, and the most recent reference with a citation burst was observed in 2017. Among these 25 references, six references (24%) had a duration until 2021. The reference with the strongest burstness (strength = 128.73, from 2014 to 2019) was published in *Cell* by Yang, Wan Seok et al., followed by the reference published in *Cell* by Dixon, Scott J et al. (strength = 88.88, from 2013 to 2017) and *Nature Cell Biology* by Angeli, Jose Pedro Friedmann et al. (strength = 84.29, from 2015 to 2019).

**TABLE 7:** Top 20 ferroptosis-related publications in cancer with the most citations (up to 19 December 2021).

Rank	Title	Type	First author	Journal	Year	Citation
1	Ferroptosis: An Iron-Dependent Form of Nonapoptotic Cell Death	Article	Scott J Dixon	Cell	2012	3,009
2	Molecular mechanisms of cell death: recommendations of the Nomenclature Committee on Cell Death 2018	Review	Lorenzo Galluzzi	Cell Death and Differentiation	2018	1,645
3	Regulation of Ferroptotic Cancer Cell Death by GPX4	Article	Wan Seok Yang	Cell	2014	1,554
4	Ferroptosis: A Regulated Cell Death Nexus Linking Metabolism, Redox Biology, and Disease	Review	Brent R Stockwell	Cell	2017	1,444
5	Regulated necrosis: the expanding network of non-apoptotic cell death pathways	Review	Tom Vanden Berghe	Nature Reviews Molecular Cell Biology	2014	934
6	Inactivation of the ferroptosis regulator Gpx4 triggers acute renal failure in mice	Article	Jose Pedro Friedmann Angeli	Nature Cell Biology	2014	925
7	The role of iron and reactive oxygen species in cell death	Review	Scott J Dixon	Nature Chemical Biology	2014	919
8	Ferroptosis: process and function	Review	Yangchun Xie	Cell Death and Differentiation	2016	869
9	Ferroptosis as a p53-mediated activity during tumour suppression	Article	Le Jiang	Nature	2015	811
10	Ferrootosis: Death by Lipid Peroxidation	Review	Wan Seok Yang	Trends in Cell Biology	2016	724
11	ACSL4 dictates ferroptosis sensitivity by shaping cellular lipid composition	Article	Sebastian, Doll	Nature Chemical Biology	2017	681
12	Oxidized arachidonic and adrenic PEs navigate cells to ferroptosis	Article	Valerian E Kagan	Nature Chemical Biology	2017	581
13	Pharmacological inhibition of cystine-glutamate exchange induces endoplasmic reticulum stress and ferroptosis	Article	Scott J Dixon	elife	2014	575
14	Glutaminolysis and Transferrin Regulate Ferroptosis	Article	Minghui Gao	Molecular Cell	2015	558
15	Lipid peroxidation in cell death	Article	Michael M Gaschler	Biochemical and Biophysical Research Communications	2017	513
16	Simultaneous Fenton-like Ion Delivery and Glutathione Depletion by MnO2-Based Nanoagent to Enhance Chemodynamic Therapy	Article	Li-Sen Lin	Angewandte Chemie-International Edition	2018	510
17	Activation of the p62-Keap1-NRF2 pathway protects against ferroptosis in hepatocellular carcinoma cells	Article	Xiaofang Sun	Hepatology	2016	508
18	Autophagy promotes ferroptosis by degradation of ferritin	Article	Wen Hou	Autophagy	2016	485
19	Dependency of a therapy-resistant state of cancer cells on a lipid peroxidase pathway	Article	Vasanthi S Viswanathan	Nature	2017	479
20	Peroxidation of polyunsaturated fatty acids by lipoxygenases drives ferroptosis	Article	Wan Seok Yang	PNAS^a^	2016	477

a PNAS: proceedings of the national academy of sciences of the United states of america.

### Analysis of Hotspots and Frontiers

**FIGURE 5:**
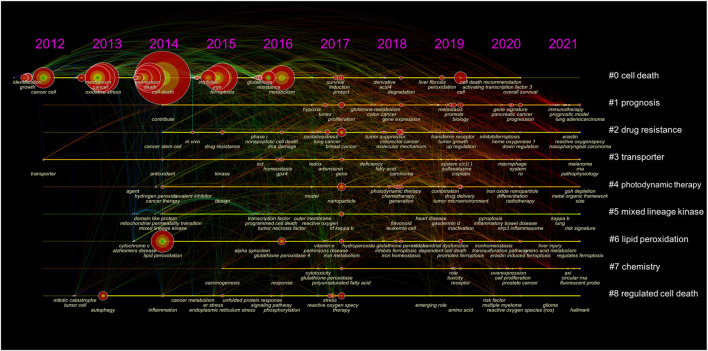
The timeline view of keywords. The nodes represented keywords (the larger circle, the more frequency of occurrence). Clusters’ labels were listed on the right.

To investigate the research hotspots in the field of ferroptosis in cancer, all keywords (including author keywords and keywords plus) were extracted from all the 1833 publications for Co-occurrence analysis on VOSviewer software. Table 8 shows the top 20 high-frequency keywords. Excluding “ferroptosis” (1,192 times), the most frequently appeared keywords (over 300 times) were “cell death” (434 times), “cancer” (360 times), “apoptosis” (335 times), “iron” (321 times), and “oxidative stress” (321 times). We used Citespace software to cluster the keywords and draw a timeline for keywords after clustering (Figure 5). Total 9 clusters were formed: #0 cell death; #1 prognosis; #2 drug resistance; #3 transporter; #4 photodynamic therapy; #5 mixed-lineage kinase; #6 lipid peroxidation; #7 chemistry; and #8 regulated cell death.

**TABLE 8:** Top 20 keywords related to the field of ferroptosis in cancer.

Rank	Keyword	Occurrence	Rank	Keyword	Occurrence
1	ferroptosis	1,192	11	cancer-cells	180
2	cell-death	434	12	Autophagy	178
3	cancer	360	13	Activation	175
4	apoptosis	335	14	Inhibition	151
5	iron	321	15	lipid-peroxidation	148
6	oxidative stress	321	16	Resistance	137
7	death	287	17	gpx4	112
8	metabolism	263	18	Cells	109
9	expression	246	19	Glutathione	100
10	mechanisms	200	20	Growth	98

**FIGURE 6:**
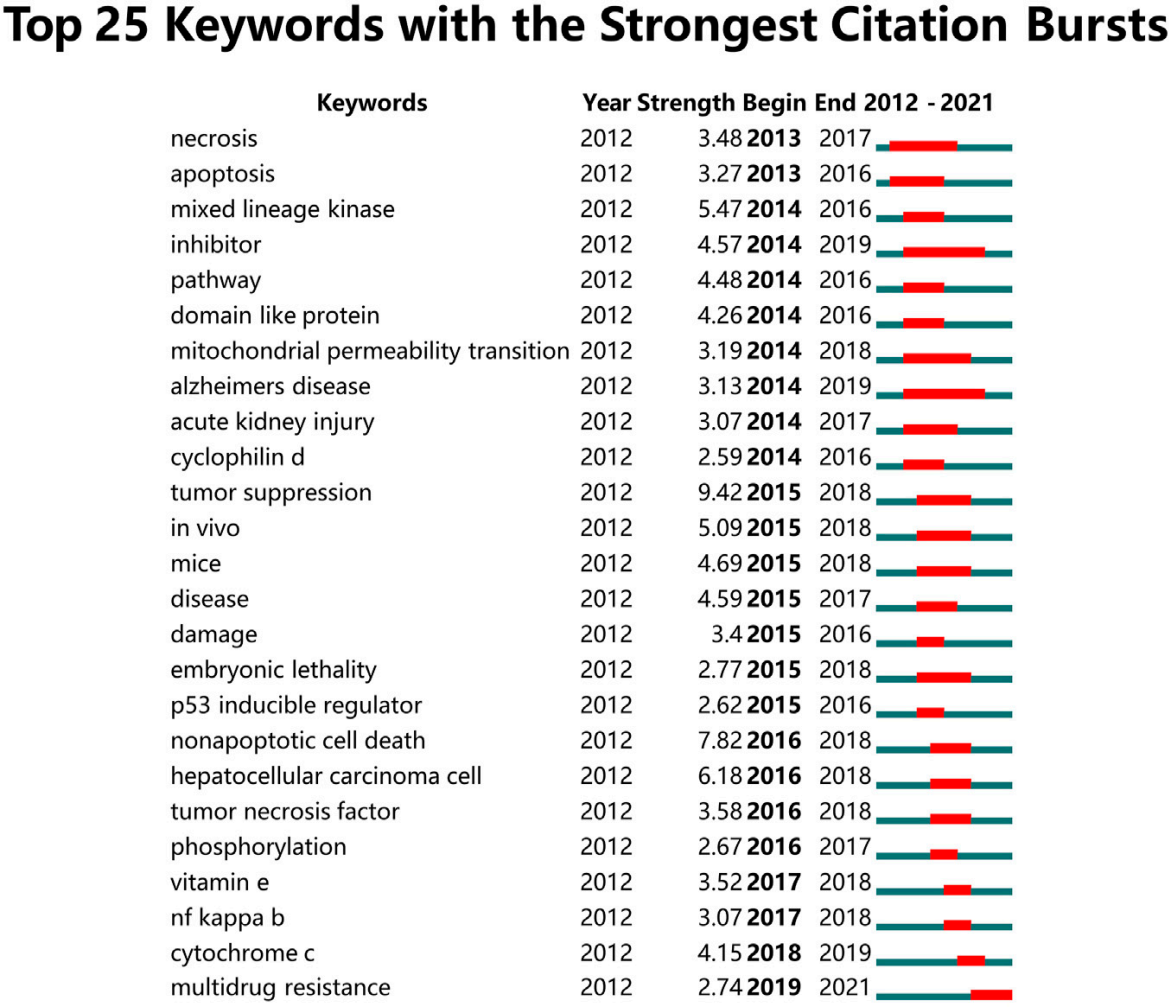
Top 25 keywords with the strongest citation bursts involved in ferroptosis in cancer. (Sorted by the beginning year of burst.)

To investigate the research frontiers, we used Citespace software to detect keywords burst (Figure 6). A red line indicates that the corresponding terms frequently appeared during this period, and a blue line represents relatively being used infrequently. Among the top 25 keywords with strong burst strength, “tumor suppression”, with a strength of 9.42, was ranked first, followed by “nonapoptotic cell death” (7.82) and “hepatocellular carcinoma cell” (6.18). Notably, “multidrug resistance” had a duration until 2021 and four keywords (“inhibitor”, “Alzheimer's disease”, “necrosis”, and “mitochondrial permeability transition”) had a duration not less than 5 years.

## Discussion

The cell is the basic unit of life. The balance between cell proliferation and cell death is therefore of critical importance for multicellular organisms, including human beings. Ferroptosis was the most recently coined form of regulated cell death, following the discovery of apoptosis (Kerr et al., 1972), necrosis (Galluzzi et al., 2017), and pyroptosis (Galluzzi et al., 2018; Hu et al., 2021). In the past 10 years, multiple roles of ferroptosis in health and disease have been confirmed (Dixon and Stockwell, 2019;Han et al., 2020;Li et al., 2020;van Swelm et al., 2020;Chen et al., 2021c;Zhang et al., 2021b). The number of publications related to ferroptosis was growing exponentially, especially in the field of cancer research (Figure 1; Table 1) (Chen et al., 2021b; Jiang et al., 2021). As a rapidly evolving research area, it was of great challenge to gain a systematic understanding of developments, especially for those who are new to this field. To get a better understanding of the research trends in the field of ferroptosis in cancer, we conducted this study.

Previously, several bibliometric analysis studies had been conducted on ferroptosis (Zhang J. et al., 2021; Wu et al., 2021; Xiong et al., 2021). However, all these studies were set to a timeline limited to 2020 and no bibliometric studies have provided a global landscape of the ferroptosis research in cancer. In this study, a total of 1833 publications on ferroptosis in cancer were identified in the Web of Science Core Collection database from 2012 to 2021. To our surprise, more than 800 publications have been produced in 2021, accounting for almost half of all publications produced over the last 10 years. Previously, several studies have been conducted on bibliometric analysis of ferroptosis in all fields, including 1,285 publications (Wu et al., 2021), 1,363 publications (Xiong et al., 2021), and 1,267 publications (Zhang J. et al., 2021), respectively, with the timeline limited to 2020. To our best knowledge, the current study was the first comprehensive bibliometric study conducted on ferroptosis in the field of cancer research.

Generally, the number of publications in a research area can reflect productivity (Durieux and Gevenois, 2010), and the number of citations was used to represent the impact (Muniz et al., 2018). They are two main bibliometric indicators to evaluate the level of research (Seglen, 1997). Based on the observed (Figure 1) and predicted (Supplementary Figure S2) trends of publications and citations from 2012 to 2021, we can pronounce that the field of ferroptosis in cancer has entered a rapid growth period. More than 60 countries/regions had published papers on ferroptosis (Supplementary Table S2), indicating that the research on ferroptosis in cancer has attracted attention from all over the world. China and United States had the most publications and citations, respectively (Table 2). This indicated that Chinese researchers are of great interest in the research of ferroptosis in cancer and United States was in a dominant position in this field. Interestingly, the top 4 countries (China, United States, Germany, and Japan) with the highest number of publications (Table 2) were also the top 4 countries ranked by gross domestic product (GDP) (https://datacatalog.worldbank.org/dataset/gdp-ranking, accessed on 16 November 2021). This suggested that economic development may affect academic research, especially for an active field like ferroptosis in cancer.

**FIGURE 7:**
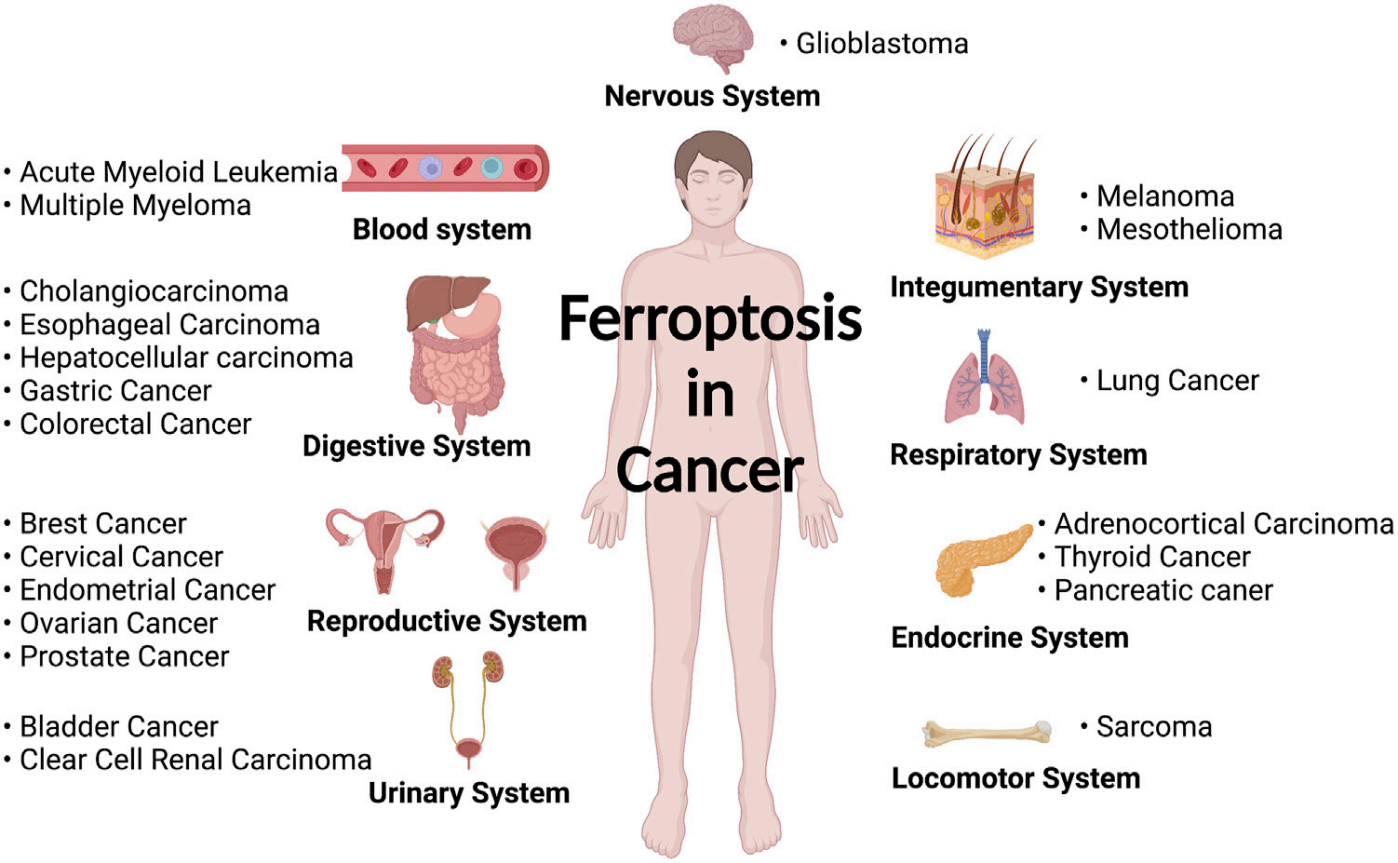
Diseases related to ferroptosis in various human body systems (created with https://biorender.com/).

Throughout the 10 years, *Biochemical and Biophysical Research Communications* (n = 54) and *Frontiers in Oncology* (n = 54) published the highest number of manuscripts in the field of ferroptosis in cancer (Table 5). Despite having a relatively low impact (located in the JCR Q2 region), these two journals made a notable contribution to the development of ferroptosis in cancer research. Among the top 10 most cited publications (Dixon et al., 2012; Angeli et al., 2014; Dixon and Stockwell, 2014; Vanden Berghe et al., 2014; Yang et al., 2014; Jiang et al., 2015; Xie et al., 2016; Yang and Stockwell, 2016; Stockwell et al., 2017; Galluzzi et al., 2018), six were reviews, indicating the important role of this type of literature. As listed in Table 7, the top 20 articles are considered fundamental to ferroptosis in cancer research. We suggested reading these papers to get an in-depth knowledge of the field. Based on the publications collected in this study, we summarized all the currently known cancer types associated with ferroptosis in Figure 7.

Although ferroptosis contributed to a plethora of human diseases, the roles of ferroptosis in cancer have been by far the most extensively studied. Overall, cancer cells have been shown to have a more active metabolism, higher reactive oxygen species load, and higher iron demand than normal cells (Hassannia et al., 2019; Jiang et al., 2021). These characteristics rendered the ferroptosis pathway a promising target for cancer treatment. In addition, recent discoveries indicated that perturbation in several cancer-related pathways (such as RAS, TP53, NFE2L2, autophagy, and hypoxia) is linked to an acquired sensitivity to ferroptosis (Chen et al., 2021b). Currently, a list of clinical trials of potential ferroptosis inducers in oncology is ongoing, including SLC7A11 inhibitors (Sorafenib and Sulfasalazine), GPX4 inhibitors (Altretamine and Withaferin A), and iron activators (Neratinib, Salinomycin, Lapatinib) (Chen et al., 2021b). The combination of proferroptotic drugs with other anticancer therapies (such as immunotherapy or radiotherapy) may be a potential strategy to overcome resistance to traditional cancer therapies.

Multidrug resistance was defined as a complex phenotype characterized by developing resistance to a broad range of structurally unrelated anticancer drugs (Gottesman, 2002). It was a significant biomedical and clinical problem leading to treatment failure in cancer (Assaraf et al., 2019). The most common underlying mechanism of multidrug resistance was the role of ATP-binding cassette (ABC) transporters in drug efflux from cancer cells. Recently, Cao et al. showed that disruption of the ABC-family transporter multidrug resistance protein 1 (MRP1) prevents glutathione efflux from the cell and strongly inhibits ferroptosis, highlighting the role of ferroptosis in overcoming cancer multidrug resistance (Cao et al., 2019). Furthermore, other groups also provided evidence that triggering ferroptosis with compounds shows great potential in cancer therapy by bypassing the multidrug resistance effect (Mbaveng et al., 2018; Gao et al., 2019; Zhou et al., 2019; Du et al., 2021). Nanoparticle drug delivery, chemical optoepigenomics, and polymeric photothermal agents were emerging tools of great advantage in ferroptosis therapy to overcome multidrug resistance (Shi et al., 2018; Zhang X. K. et al., 2021; Fu et al., 2021). Of note, in the present study, the top three largest clusters were labeled “cell death, “prognosis”, and “drug-resistance” in the clustering analysis of keywords (Figure 5), and “multidrug resistance” were the only keyword that had duration until 2021 (Figure 6). Together, these results suggested that future research on novel strategies to overcome drug or multidrug resistance may be the frontier in the field of ferroptosis in cancer.

The present study was not without limitations. Firstly, this study queried only the Web of Science Core Collection database. High-quality papers published in journals that are not included in the Web of Science Core Collection database may be missed. The combination of Web of Science with other databases, such as PubMed, Google Scholar, and Scopus, would enable a more robust bibliometric analysis. Secondly, this study also comes with certain limitations inherent in CiteSpace and VOSviewer software. For instance, some keywords in the papers maybe not be included in the analysis due to the incomplete keyword extraction method and the clustering analysis based on only the main information (not the full text). Thirdly, authors, institutions, or keywords may have different expression types, bias may exist.

## Conclusion

Overall, the bibliometric analysis of this study offers comprehensive information about the publication performance of ferroptosis in cancer. Our results showed that research on ferroptosis is flourishing in the field of cancer. Ferroptosis has aroused great interest in the community of cancer research all over the world. Although in its infancy, triggering ferroptosis holds great potential for the development of cancer treatment, especially for overcoming multidrug resistance. Future research on novel strategies to overcome drug or multidrug resistance may be the frontier in the field of ferroptosis in cancer.

## Data Availability

The original contributions presented in the study are included in the article/Supplementary Material, further inquiries can be directed to the corresponding author.
