# Supplementary figures and images for: The Research Landscape of Ferroptosis in Cancer: A Bibliometric Analysis
Source: Front Cell Dev Biol. 2022 May 25;10:841724. doi: 10.3389/fcell.2022.841724 (PMC9174675; doi:10.3389/fcell.2022.841724)

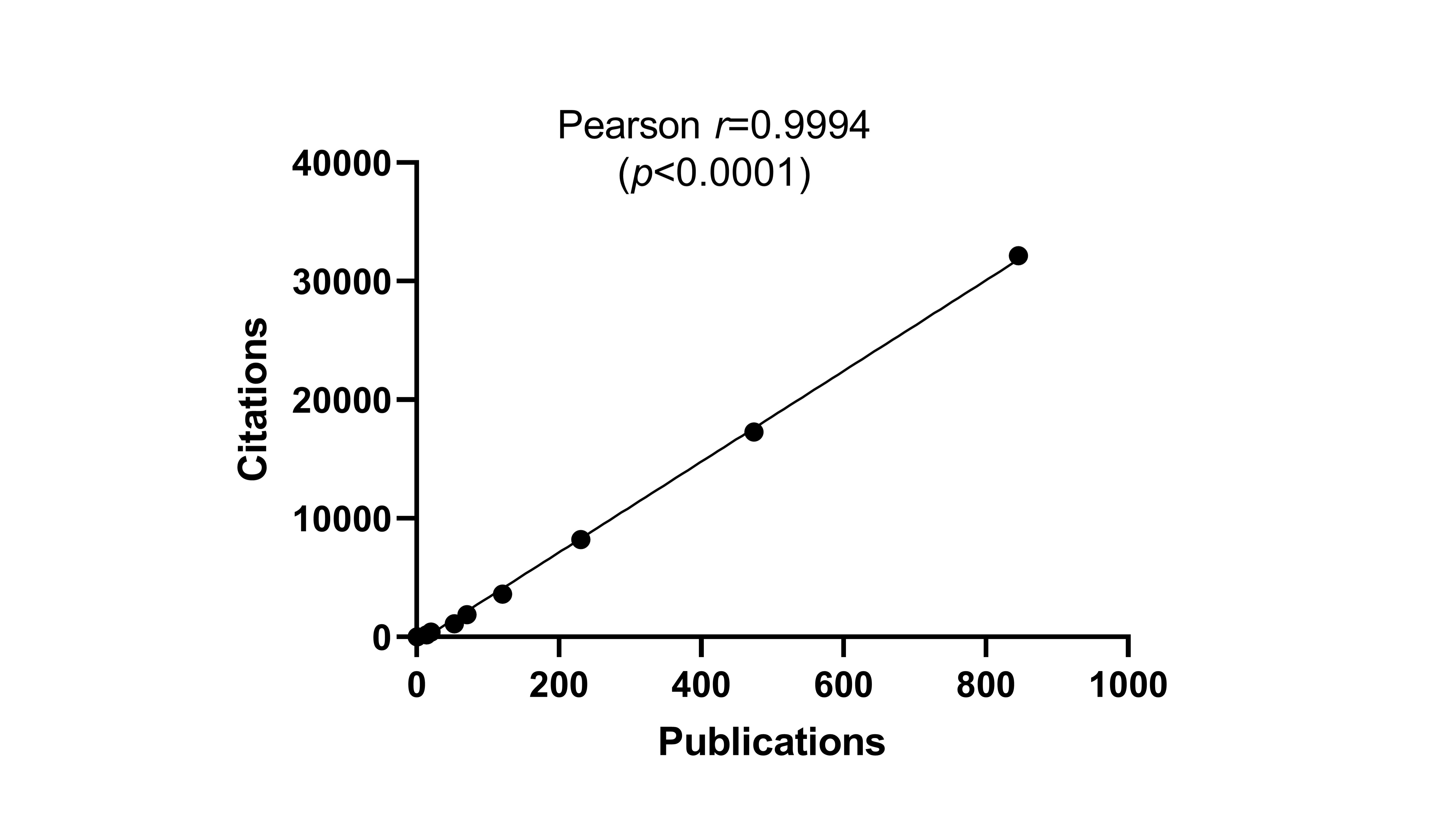

Supplement: Supplementary file 3 [file Image3.JPEG]

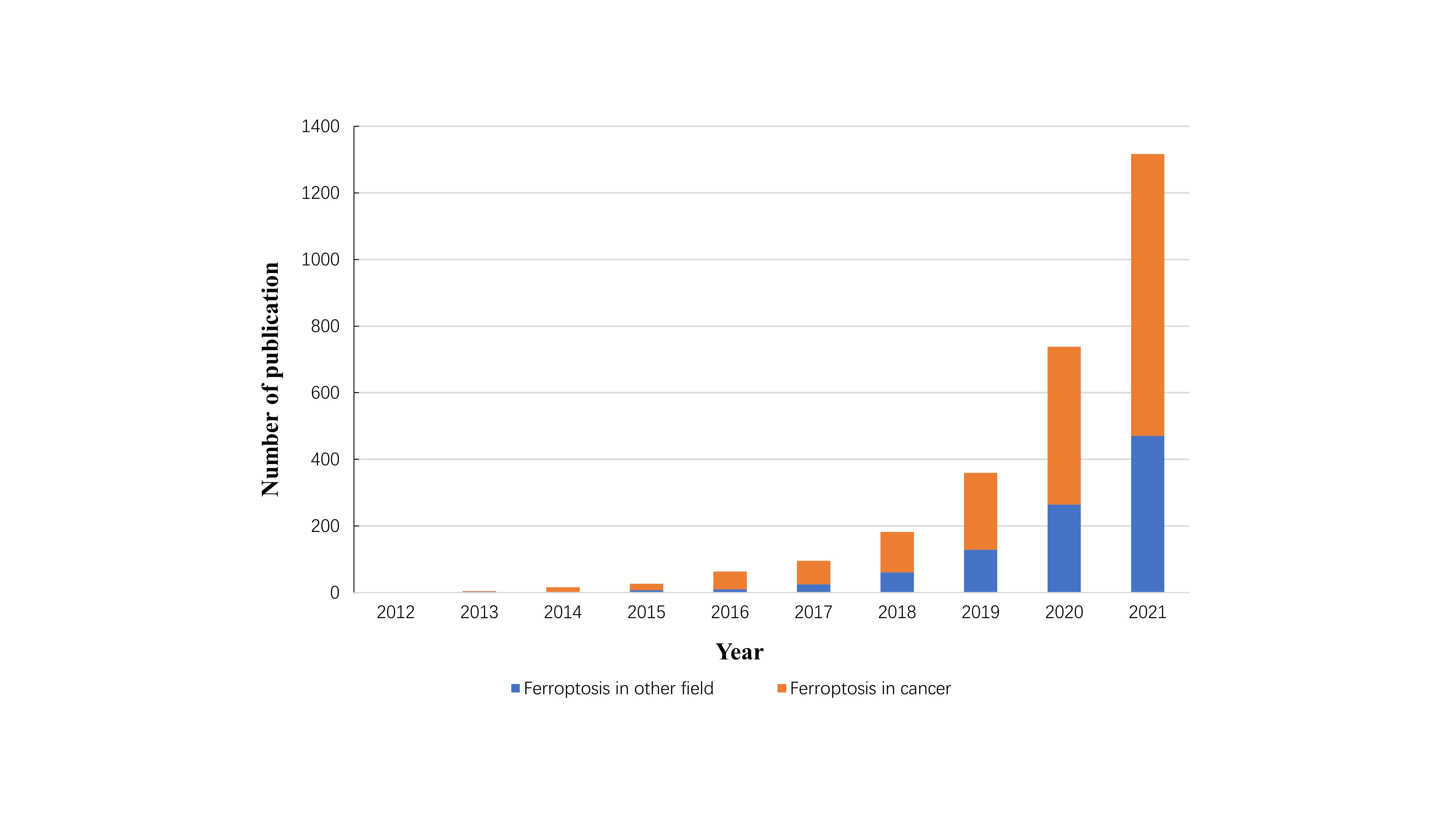

Supplement: Supplementary file 5 [file Image1.JPEG]

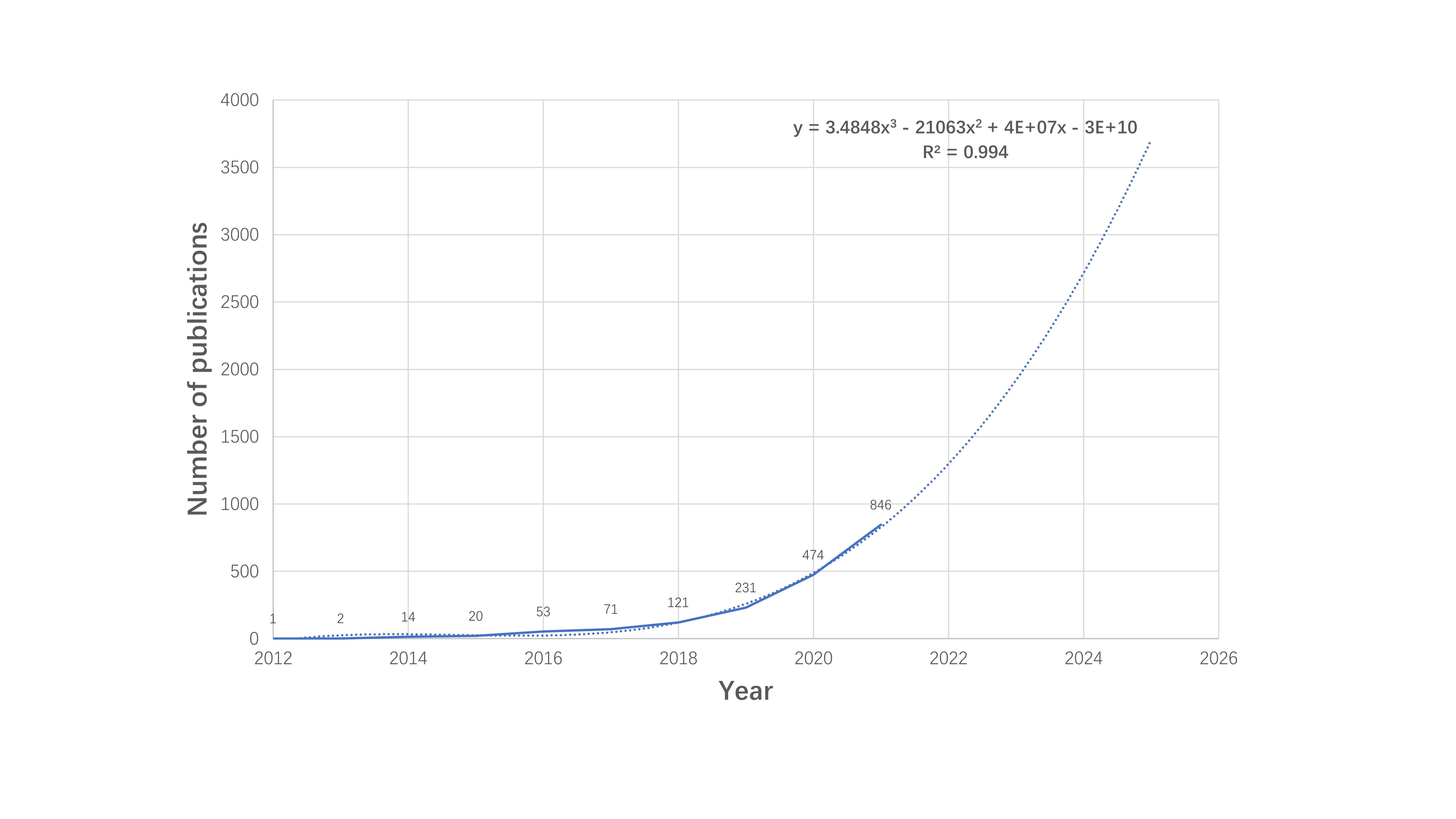

Supplement: Supplementary file 6 [file Image2.JPEG]
